# HLA-DRB1*∗*16:01 and HLA-DQB1*∗*05:02 Alleles Influence the Susceptibility and Progression of Cutaneous Malignant Melanoma

**DOI:** 10.1155/2021/3801143

**Published:** 2021-09-30

**Authors:** Xu Wang, Francisco Almazan, Yoel Genaro Montoyo-Pujol, Antonia Martin-Casares, Aurelio Martin, Teresa Cabrera, Miguel Angel López-Nevot

**Affiliations:** ^1^Servicio de Análisis Clínicos e Inmunología, Hospital Universitario Virgen de las Nieves, Avda. Fuerzas Armadas s/n, Granada 18014, Spain; ^2^Programa de Doctorado en Biomedicina, Universidad de Granada, Granada, Spain; ^3^Servicio de Dermatología, Hospital Universitario Clinico San Cecilio (PTS), Av. de la Investigación, s/n, Granada 18016, Spain; ^4^Servicio de Anatomía Patológica, Hospital Universitario Virgen de las Nieves, Avda. Fuerzas Armadas s/n, Granada 18014, Spain; ^5^Departamento Bioquímica, Biología Molecular e Inmunología III, Universidad de Granada, Granada, Spain; ^6^Instituto de Investigación Biosanitaria I ibs. GRANADA, Avda de Madrid 15, Pabellón de Consultas Externas 2, Granada 18012, Spain

## Abstract

**Background:**

The influence of HLA class I and II loci on the susceptibility to melanoma remains an area of intense debate. This study aimed to examine whether the HLA system was related to melanoma susceptibility and prognosis in a southern Spanish population.

**Methods:**

In this study, HLA class I and class II genotyping were performed using polymerase chain reaction sequence-specific oligonucleotides (PCR-SSO) in 237 Spanish melanoma patients and 636 ethnically matched controls. Data were analyzed according to the clinical characteristics of the defined subgroups.

**Results:**

Compared to the control group, DRB1*∗*16:01 (4% vs. 1.3%, *p*=0.001, Pc = 0.035, OR = 3.28) and DQB1*∗*05:02 (4.9% vs. 2%, *p*=0.001, Pc = 0.017, OR = 2.54) were positivity associated with the susceptibility to melanoma. Both DRB1*∗*16:01 (5.4% vs. 1.3%, *p*=0.001, Pc = 0.035, OR = 4.46) and DQB1*∗*05:02 (6.5% vs. 2%, *p*=0.001, Pc = 0.017, OR = 3.44) also showed a positive correlation with Breslow thickness >1.5 mm, most notably at an early age of diagnosis (≤58 years), DRB1*∗*16:01 (4.2% vs. 1.3%, *p*=0.001, Pc = 0.035, OR = 3.41) and DQB1*∗*05:02 (5.4% vs. 2%, *p*=0.002, Pc = 0.034, OR = 2.86).

**Conclusion:**

These findings established HLA-DRB1*∗*16:01 and HLA-DQB1*∗*05:02 loci as melanoma risk factors in the southern Spanish population.

## 1. Introduction

Malignant melanoma is characterized by a variable clinical outcome due to the complex interactions of human leukocyte antigens (HLAs), antigen-specific cytolytic T cells (CTLs), and natural killer (NK) cells [1–3]. During immune surveillance, HLA class I molecules participate in tumor cell recognition for CD8+ T-cell-mediated clearance [4, 5], whilst HLA class II molecules stimulate CD4+ T cells to combat melanoma progression [6]. It is, therefore, speculated that the susceptibility to melanoma is closely associated with specific HLA genes [7].

Studies have reported the association between melanoma and HLA genes with variable outcomes. Ichimiya et al. observed an increase in the HLA-B13 antigen and a decrease in the frequency of the HLA-B61 antigen in Japanese melanoma patients compared to healthy controls [8]. Schachter et al. reported an increase in the frequency of HLA-A24, -A26, and -B38 antigens in Jewish melanoma patients compared with control cases [9]. Luongo et al. described the association of HLA-A*∗*01 with less aggressive disease, which negatively associated with HLA-B*∗*13 and HLA-B*∗*44 in Italian melanoma patients [10]. In contrast, Campillo et al. observed no association with any HLA class I alleles in southeastern Spanish patients and described an association for patients homozygous for group C2 (lysine at position 80) of HLA-C recognized by the killer immunoglobulin-like receptors (KIRs) with metastatic progression. A negative association between group C1 (asparagine at position 80) and the susceptibility to melanoma progression and metastasis have also been reported [11].

Conflicting results have also been reported in studies investigating the role of HLA class II genes in melanoma susceptibility. Barger et al. described the first positive association between the HLA-DR4 allele and melanoma susceptibility in the Caucasian American population of Alabama, in which the HLA-DR3 allele favorably influenced prognosis [12]. HLA-DQB1*∗*03:01 was more frequent in melanoma patients than in a control series of 45 patients (Lee et al., 1994) and represented a risk factor for local recurrence and the generation of regional and distant metastases in 259 American patients (Lee et al., 1996) [13, 14]. An increase in the frequency of HLA-DQB1*∗*03:01 was observed in Italian melanoma patients but without statistical significance [15]. In the English population, similar results were obtained for DQB1*∗*03:01 in terms of the risk of melanoma, but the most frequent allele in patients was HLA-DQB1*∗*03:03 as opposed to HLA-DQB1*∗*03:01 [16]. Conversely, Lulli et al. observed a decrease in the frequency of HLA-DQB1*∗*03 and all haplotypes that included three alleles within the family DQB1*∗*03:01, DQB1*∗*03:02, and DQB1*∗*03:03, although this study included only 53 melanoma patients [17]. Luongo et al. also observed no association between HLA class II genes and the susceptibility to melanoma [10]. In two Spanish studies performed in Valencia (Nagore et al. and Planelles et al.) and Murcia (Campillo et al.) and in a cohort of Japanese patients, no association was observed between the HLA-DRB1 and HLA-DQB1 alleles and human malignant melanoma [11, 18, 19].

In this study, we expanded the number of patients (*n* = 237) and control subjects (*n* = 636) to define the relationship more accurately between melanoma susceptibility and the frequency of HLA genes.

## 2. Materials and Methods

### 2.1. Patients/Controls

Blood samples were obtained from 237 patients diagnosed with melanoma. Tumor staging was performed from biopsy samples according to the criteria of the American Joint Committee on Cancer TNM staging system (AJCC 2018) at the Dermatology Service of Virgen de las Nieves University Hospital of Granada, Spain. Exclusion criteria were as follows: cardiac disease, autoimmune disease, and diabetes mellitus. Control subjects included 637 age- and gender-matched healthy donors obtained from the blood bank of Granada. All healthy donors had no indication of immune-related disease. Blood samples were collected from 2016 to 2019. Patient blood samples were obtained prior to primary melanoma excision. All subjects provided written informed consent. The study protocol was approved by the hospital institutional review board and ethical committee. The clinicopathological characteristics of the patients are summarized in Table 1.

### 2.2. DNA Extraction and Determination of HLA Class I and II Genotypes

Genomic DNA was extracted from peripheral venous blood leukocytes using commercial QIAamp DNA Blood Maxi Kits (QIAGEN, Hilden, Germany) according to the manufacturer's instructions. High-resolution genotyping of HLA class I (A, B, and C) and class II (DRB1 and DQB1) loci typing was performed using the LABType sequence-specific oligonucleotide typing test (One Lambda, Canoga Park, CA, USA). Target DNA was amplified by PCR using HLA locus-specific primers, followed by hybridization with allele-specific oligodeoxynucleotides coupled with fluorescent phycoerythrin-labeled microspheres. Allele genotyping was determined based on the reaction pattern of the microspheres using the LABScan 100 system (Luminex xMAP, Austin, TX, USA). HLA alleles were assigned using HLA Fusion software (One Lambda). HLA typing was presented as four digits, taking into account the common and well-defined alleles (CWD).

### 2.3. Statistical Analysis

SPSS.20 (SPSS Inc., Chicago, IL, USA) was used for all data analyses. Differences in allele frequencies between melanoma patients and the control groups were evaluated using the chi-square method (*χ*^2^, Mantel–Haenszel) using 3 × 2 or 2 × 2 contingency tables. Fisher's exact test was applied for predicted values ≤5. The strength of an association was calculated using the odds ratio (OR) according to the Woolf method with Haldane modifications for 95% confidence intervals (CIs). *p* values ≤0.05 were considered statistically significant. Pc (i.e., corrected *p*) was the *p* value of the Bonferroni correction. Bonferroni correction was performed through the multiplication of all alleles assessed within each locus.

## 3. Results

### 3.1. HLA Frequency in Melanoma Patients

The frequencies of HLA-A, -B, and -C alleles in 237 melanoma patients were compared with 637 normal control subjects of the same ethnic origin (Table 2). Within the patient cohort, 30 alleles for HLA-A, 49 alleles for HLA-B, and 25 alleles for HLA-C were detected. No significant differences in the frequency of HLA-A, -B, and -C alleles were observed between groups, although some were of higher number in melanoma patients compared with healthy controls (HLA-A*∗*02:05: 3% vs. 1.1%, *p*=0.006, Pc = 0.18, and HLA-B*∗*35:03: 3.6% vs. 1.5%, *p*=0.006, Pc = 0.29).

For HLA class II, 35 HLA-DRB1 and 17 HLA-DQB1 alleles were identified. Only the frequencies of DRB1*∗*16:01 (4% vs. 1.3%, *p*=0.001, Pc = 0.035, OR = 3.28) and DQB1*∗*05:02 (4.9% vs. 2%, *p*=0.001, Pc = 0.017, OR = 2.54) significantly increased in melanoma patients. Previous studies reported that HLA-DQB1*∗*03:01, -DQB1*∗*03:02, -DQB1*∗*03:03, and -DQB1*∗*05:01 alleles were associated with melanoma, but no association of these alleles between melanoma patients and healthy controls was observed in our cohort (DQB1*∗*03:01: 18.6% vs. 17.9%, *p*=0.757; DQB1*∗*03:02: 5.9% vs. 9.2%, *p*=0.027, Pc = 0.459; DQB1*∗*03:03: 2.5% vs. 3.4%, *p*=0.366; DQB1*∗*05:01: 13.1% vs. 14.4%, *p*=0.484, Table 3).

### 3.2. HLA Frequency and Melanoma Prognosis

Breslow thickness is a strong predictor of patient survival as it defines the depth of tumor invasion from the granular stratum to the deepest penetrating melanoma cells. In this study, patient and control groups were defined as having a Breslow thickness of ≤1.5 mm (low risk, *n* = 144) or >1.5 mm (intermediate or high risk, *n* = 93), as described by Planelles et al. in Spanish melanoma patients [19]. These analyses revealed that DRB1*∗*16:01 (5.4% vs. 1.3%, *p*=0.001, Pc = 0.035, OR = 4.46) and DQB1*∗*05:02 (6.5% vs. 2%, *p*=0.001, Pc = 0.017, OR = 3.44) were of significantly higher frequency in the subgroup with Breslow thickness >1.5 mm compared with healthy controls. No significant differences were observed between HLA-A, -B, and -C alleles in the subgroup with Breslow thickness >1.5 mm. Neither HLA-A, -B, and -C nor HLA-DRB1 and -DQB1 showed significant differences in the subgroup with a Breslow thickness of ≤1.5 mm.

To explore the correlation between the HLA allele frequency and the age of diagnosis, Planelles et al. used a cutoff of 30 years [19]. This grouping standard was not applicable to our patient cohort given their older age (only 14 cases aged ≤30 years; median age: 58 years). Melanoma patients were therefore divided into two subgroups according to their age of diagnosis: ≤58 years (*n* = 119) or >58 years (*n* = 118). DRB1*∗*16:01 (4.2% vs. 1.3%, *p*=0.001, Pc = 0.035, OR = 3.41) and DQB1*∗*05:02 (5.4% vs. 2%, *p*=0.002, Pc = 0.034, OR = 2.86) (Table 4) were of a significantly higher frequency in the subgroup with the age of diagnosis ≤58 years compared to healthy controls. No significant differences in HLA-A, -B, and -C alleles were observed in subjects aged >55 years.

There was no significant difference in frequency between patients with a Breslow thickness of ≤1.5 mm and >1.5 mm nor between those aged ≤58 years and >58 years.

To study the differences in the frequency of HLA alleles according to the melanoma type, 154 cases of superficial spreading melanoma (SSM), 54 cases of nodular melanoma (NM), 19 cases of lentigo maligna melanoma (LMM), and 10 cases of acral lentiginous melanoma (ALM) were compared. Following these analyses, no significant differences were observed between melanoma and control groups. The frequency of HLA class I and HLA class II antigens also did not significantly differ between the sentinel lymph node metastasis and ulceration cases.

### 3.3. HLA Homozygosity in Melanoma Patients

To further explore the correlation between HLA alleles and melanoma, we compared the homozygosity frequency of HLA-A, -B, and -C and HLA-DRB1 and -DQB1 between melanoma patients and healthy controls. Homozygosity frequency of different HLA alleles was also performed according to the clinicopathological features of each patient subgroup. No significant differences following these analyses were observed.

## 4. Discussion

In this study, we performed a comprehensive analysis of polymorphisms of HLA class I and class II alleles in southern Spanish patients with melanoma. We found that HLA-DRB1*∗*16:01 and HLA-DQB1*∗*05:02 were of a higher frequency in melanoma patients compared with healthy controls (Table 3), suggesting that these two loci may be related to the susceptibility of melanoma. Interestingly, when we investigated the association between HLA allele frequency and clinical and histological parameters, the frequency of both loci was found to be significantly increased in patients with an intermediate or high risk of Breslow thickness (Breslow thickness >1.5 mm) (Table 4). Breslow thickness is an important predictor of patient survival and a key parameter of the AJCC TNM classification for primary melanoma patients. A higher Breslow thickness indicates lower mortality and a more advanced tumor stage; for example, tumors ≤1.0 mm are designated as low risk with 10-year survival rates of 92%; tumors between 1.01 and 2.0 mm have 10-year survival rates of 80%; tumors measuring 2.01 to 4.0 mm have 10-year survival rates of 63%; tumors ≧4 mm have 10-year survival rates of 50% [20, 21]. In addition, age is also an important factor that affects the prognosis of melanoma. Many studies have shown that elderly patients with melanoma have a worse prognosis and that melanoma patients over the age of 60 are a high-risk group [22, 23]. In this study, HLA-DRB1*∗*16:01 and HLA-DQB1*∗*05:02 were significantly increased in the subgroup of patients diagnosed at an early age (age of diagnosis of ≤58 years). This indicated that HLA-DRB1*∗*16:01 and HLA-DQB1*∗*05:02 may be related to the susceptibility of melanoma and play an important role in melanoma progression.

It should be noted that, within the Spanish population, HLA-DRB1*∗*16:01 and HLA-DQB1*∗*05:02 were not the most frequent loci. HLA-DRB1*∗*16:01 had an allele frequency of 1.3% in healthy subjects compared with the 2% frequency observed for HLA-DQB1*∗*05:02. The low frequency of these two loci may explain their absence in previous studies performed in the Spanish population. Studies performed by Nagore et al. included 82 patients and 367 healthy controls, while Planelles et al. compared HLA frequency in 117 melanoma patients and 301 healthy donors. Campillo et al. performed their analyses in 174 melanoma patients and 227 ethnically matched controls [11, 18, 19]. In this study, the sample size was expanded to 237 melanoma patients and 637 healthy controls.

As both HLA-DRB1*∗*16:01 and HLA-DQB1*∗*05:02 loci have been reported as haplotypes in the Spanish population [24–26], it was not possible to identify which allele was responsible for the association or whether coexpression of both was necessary. There are two possible hypotheses for the association of the HLA-DRB1*∗*16:01-DQB1*∗*05:02 haplotype with the susceptibility to melanoma and a higher Breslow thickness of the tumor: (1) this haplotype induces the differentiation of regulatory T cells that can inhibit the cellular response to melanoma; (2) this haplotype deactivates CD4+ Th1 lymphocytes that recognize peptides generated from melanoma antigens, thereby preventing their association with antitumor CD8+ lymphocytes. It has been shown that the DQB5 molecule (including the DQB1*∗*05:02 variant) presents Melan-A/MART melanoma antigen peptides to CD4+ lymphocytes with cytotoxic capacity *in vitro*. The HLA-DRB1*∗*16:01 molecule is therefore responsible for the immunosuppressive effects observed in melanoma patients [27].

Previous studies reported that HLA-DQB1*∗*03:01, -DQB1*∗*03:02, -DQB1*∗*03:03, and -DQB1*∗*05:01 alleles are associated with melanoma [10, 13, 15, 16]. In this study, no significant frequency of these loci was observed, consistent with previous studies performed in the Spanish population [11, 18, 19]. These discrepancies may be due to ethnic differences, with previous analyses performed in American, Italian, and British populations.

We found that specific HLA class I alleles were of a higher frequency in melanoma patients compared to healthy controls. These differences were however nonsignificant following correction for multiple comparisons due to a large number of each locus. Examples include HLA-A*∗*02:05 (3% vs. 1.1%, *p*=0.006, Pc = 0.18) and HLA-B*∗*35:03 (3.6% vs. 1.5%, *p*=0.006, Pc = 0.296). These results are consistent with previous studies in the Spanish population which concluded that melanoma susceptibility is not influenced by HLA-A, -B, and -C [11].

Regarding the homozygosity frequency of HLA class I and class II, no significant differences were observed between melanoma patients and controls or upon subgroup comparisons according to clinicopathological characteristics. Planelles et al. reported an increased homozygosity rate for DQA1*∗*05:05 and DQA1*∗*03:01 compared to controls, but HLA-DQA1 was not included in our analysis. In addition, HLA-DQB1*∗*03:01 homozygous individuals were of a significantly higher frequency in red- or fair-haired patients. These grouping standards were not analyzed within this study [19].

Upon comparison between HLA allele frequency and clinical and histological parameters, no association between HLA and ulceration, sentinel lymph node biopsy, and the type of melanoma was observed, consistent with previous studies. Lee et al. reported that HLA-DQB1*∗*03:01 was of a higher frequency rate in subgroups with thicker melanomas and regional metastasis [13]. In this study, the frequency of HLA-DQB1*∗*03:01 was comparable between melanoma and control patients (17% vs. 17.9%, respectively). In contrast, HLA-DQB1*∗*05:02 (6.5% vs. 2%, *p*=0.001, Pc = 0.017, OR = 3.44) showed an increased frequency in melanoma patients with thicker Breslow. These differences may be explained by the distinct ethnical origins of the studies.

Some limitations of this study should be noted. First, HLA-DRB1*∗*16:01 and HLA-DQB1*∗*05:02 were not the most frequent alleles. Second, the sample number will be increased in future studies to provide more stable and reliable data. The relationship between HLA class II allele linkage disequilibrium and melanoma also requires assessment in future studies.

## 5. Conclusion

In summary, HLA-DRB1*∗*16:01 and HLA-DQB1*∗*05:02 showed a positive association with melanoma. In addition, both alleles were of a higher frequency in subgroups with a higher Breslow thickness and relatively early age of diagnosis. Based on these results, we support the notion that the HLA-DRB1*∗*16:01 and HLA-DQB1*∗*05:02 loci represent risk factors for melanoma development and progression.

## Figures and Tables

**Table 1:** Clinical characteristics.

Characteristic	Number	Frequency (%)
Patient	237	
Male	111	46.8
Female	126	53.2

Age median (minimum–maximum)	58 (21–85)	
≤58 years	119	50.2
>58 years	118	49.8

Histological type		
Superficial spreading melanoma (SSM)	154	64.9
Nodular melanoma (NM)	54	22.8
Lentigo maligna melanoma (LMM)	19	8
Acral lentiginous melanoma (ALM)	10	4.3

Breslow thickness		
≤1.5 mm	145	61.2
>1.5 mm	92	38.8

Ulceration		
Yes	54	22.8
No	183	77.2

Sentinel lymph node biopsy		
Not available	47	19.8
Available	190	80.2
Positive	42	22.1
Negative	148	77.9

**Table 2:** HLA class I alleles within the patient cohort.

HLA-A	Healthy donor (*N* = 1272), *n* (AF)	Melanoma patient (*N* = 474), *N* (AF)	HLA-B	Healthy donor (*N* = 1272), *n* (AF)	Melanoma patient (*N* = 474), *N* (AF)	HLA-C	Healthy donor (*N* = 1272), *n* (AF)	Melanoma patient (*N* = 474), *N* (AF)
A^*∗*^01:01	127 (0.1)	42 (0.089)	B^*∗*^07:02	117 (0.092)	31 (0.065)	C^*∗*^01:02	41 (0.032)	13 (0.027)
A^*∗*^01:02	2 (0.002)	0 (0)	B^*∗*^07:05	2 (0.002)	0 (0)	C^*∗*^02:02	63 (0.05)	36 (0.076)
A^*∗*^01:03	1 (0.001)	0 (0)	B^*∗*^07:06	5 (0.004)	0 (0)	C^*∗*^02:10	1 (0.001)	0 (0)
A^*∗*^02:01	312 (0.245)	116 (0.245)	B^*∗*^08:01	64 (0.05)	26 (0.055)	C^*∗*^03:02	5 (0.004)	0 (0)
A^*∗*^02:02	5 (0.004)	2 (0.004)	B^*∗*^13:02	21 (0.017)	10 (0.021)	C^*∗*^03:03	30 (0.024)	12 (0.025)
A^*∗*^02:05	14 (0.011)	14 (0.03)	B^*∗*^14:01	21 (0.017)	7 (0.015)	C^*∗*^03:04	22 (0.017)	10 (0.021)
A^*∗*^02:06	2(0.002)	1 (0.002)	B^*∗*^14:02	48 (0.038)	25 (0.053)	C^*∗*^04:01	184 (0.145)	57 (0.12)
A^*∗*^03:01	121 (0.095)	40 (0.084)	B^*∗*^15:01	39 (0.031)	18 (0.038)	C^*∗*^05:01	143 (0.112)	51 (0.108)
A^*∗*^03:02	7(0.006)	2 (0.004)	B^*∗*^15:03	8 (0.006)	1 (0.002)	C^*∗*^06:02	103 (0.081)	47 (0.099)
A^*∗*^11:01	94 (0.074)	36 (0.076)	B^*∗*^15:16	2 (0.002)	0 (0)	C^*∗*^07:01	159 (0.125)	65 (0.137)
A^*∗*^23:01	41 (0.032)	12 (0.025)	B^*∗*^15:17	9 (0.007)	1 (0.002)	C^*∗*^07:02	131 (0.103)	30 (0.063)
A^*∗*^24:02	135 (0.106)	38 (0.08)	B^*∗*^15:18	8 (0.006)	1 (0.002)	C^*∗*^07:04	14 (0.011)	10 (0.021)
A^*∗*^24:03	3 (0.002)	1 (0.002)	B^*∗*^18:01	131 (0.103)	42 (0.089)	C^*∗*^08:01	1 (0.001)	0 (0)
A^*∗*^25:01	22 (0.017)	10 (0.021)	B^*∗*^27:02	5 (0.004)	4 (0.008)	C^*∗*^08:02	67 (0.053)	31 (0.065)
A^*∗*^26:01	45 (0.035)	23 (0.049)	B^*∗*^27:03	3 (0.002)	0 (0)	C^*∗*^08:03	0 (0)	1 (0.002)
A^*∗*^29:01	7 (0.006)	3 (0.006)	B^*∗*^27:05	33 (0.026)	10 (0.021)	C^*∗*^12:02	24 (0.019)	6 (0.013)
A^*∗*^29:02	94 (0.074)	32 (0.068)	B^*∗*^27:07	2 (0.002)	2 (0.004)	C^*∗*^12:03	77 (0.061)	33 (0.07)
A^*∗*^30:01	32 (0.025)	10 (0.021)	B^*∗*^35:01	86 (0.068)	18 (0.038)	C^*∗*^14:02	19 (0.015)	6 (0.013)
A^*∗*^30:02	42 (0.033)	19 (0.04)	B^*∗*^35:02	15 (0.012)	6 (0.013)	C^*∗*^15:02	47 (0.037)	11 (0.023)
A^*∗*^30:04	0 (0)	1 (0.002)	B^*∗*^35:03	19 (0.015)	17 (0.036)	C^*∗*^15:04	1 (0.001)	0 (0)
A^*∗*^31:01	25 (0.02)	12 (0.025)	B^*∗*^35:08	11 (0.009)	4 (0.008)	C^*∗*^15:05	7 (0.006)	2 (0.004)
A^*∗*^32:01	43 (0.034)	18 (0.038)	B^*∗*^37:01	14 (0.011)	13 (0.027)	C^*∗*^16:01	96 (0.075)	32 (0.068)
A^*∗*^33:01	29 (0.023)	15 (0.032)	B^*∗*^38:01	32 (0.025)	16 (0.034)	C^*∗*^16:02	19 (0.015)	8 (0.017)
A^*∗*^34:02	6 (0.005)	0 (0)	B^*∗*^39:01	14 (0.011)	6 (0.013)	C^*∗*^16:04	0 (0)	1 (0.002)
A^*∗*^66:01	6 (0.005)	7 (0.015)	B^*∗*^39:06	4 (0.003)	3 (0.006)	C^*∗*^17:01	18 (0.014)	8(0.017)
A^*∗*^68:01	35 (0.028)	9 (0.019)	B^*∗*^40:01	21 (0.017)	10 (0.021)			
A^*∗*^68:02	9 (0.007)	8 (0.017)	B^*∗*^40:02	14 (0.011)	5 (0.011)			
A^*∗*^69:01	4 (0.003)	2 (0.004)	B^*∗*^40:06	3 (0.002)	1 (0.002)			
A^*∗*^74:01	2 (0.002)	0 (0)	B^*∗*^41:01	12 (0.009)	9 (0.019)			
A^*∗*^80:01	7 (0.006)	0 (0)	B^*∗*^41:02	3 (0.002)	2 (0.004)			
			B^*∗*^42:01	0 (0)	1 (0.002)			
			B^*∗*^44:01	2 (0.002)	0 (0)			
			B^*∗*^44:02	70 (0.055)	31 (0.065)			
			B^*∗*^44:03	123 (0.097)	40 (0.084)			
			B^*∗*^44:05	1 (0.001)	1 (0.002)			
			B^*∗*^45:01	20 (0.016)	4 (0.008)			
			B^*∗*^47:01	3 (0.002)	0 (0)			
			B^*∗*^48:01	1 (0.001)	1 (0.002)			
			B^*∗*^49:01	33 (0.026)	19 (0.04)			
			B^*∗*^50:01	36 (0.028)	16 (0.034)			
			B^*∗*^50:02	4 (0.003)	1 (0.002)			
			B^*∗*^51:01	112 (0.088)	42 (0.089)			
			B^*∗*^51:08	5 (0.004)	3 (0.006)			
			B^*∗*^52:01	20 (0.016)	4 (0.008)			
			B^*∗*^53:01	15 (0.01)	5 (0.011)			
			B^*∗*^55:01	9 (0.007)	3 (0.006)			
			B^*∗*^56:01	3 (0.002)	1 (0.002)			
			B^*∗*^57:01	29 (0.023)	8 (0.017)			
			B^*∗*^58:01	16 (0.013)	2 (0.004)			

AF: allele frequency; *n*: number of observed alleles.

**Table 3:** Most frequent HLA class II alleles with the patient cohort.

HLA-DRB1	Healthy donor (*N* = 1272), *n* (AF)	Melanoma patient (*N* = 474), *N* (AF)	HLA-DQB1	Healthy donor (*N* = 1272), *n* (AF)	Melanoma patient (*N* = 474), *N* (AF)
DRB1^*∗*^01:01	103 (0.081)	36 (0.076)	DQB1^*∗*^02:01	149 (0.117)	58 (0.122)
DRB1^*∗*^01:02	41 (0.032)	14 (0.03)	DQB1^*∗*^02:02	176 (0.138)	76 (0.16)
DRB1^*∗*^01:03	12 (0.009)	4 (0.008)	DQB1^*∗*^03:01	228 (0.179)	88 (0.186)
DRB1^*∗*^03:01	148 (0.116)	57 (0.12)	DQB1^*∗*^03:02	117 (0.092)	28 (0.059)
DRB1^*∗*^04:01	36 (0.028)	16 (0.034)	DQB1^*∗*^03:03	43 (0.034)	12 (0.025)
DRB1^*∗*^04:02	24 (0.019)	4 (0.008)	DQB1^*∗*^03:04	3 (0.002)	1 (0.002)
DRB1^*∗*^04:03	37 (0.029)	2 (0.004)	DQB1^*∗*^03:05	4 (0.003)	1 (0.002)
DRB1^*∗*^04:04	32 (0.025)	15 (0.032)	DQB1^*∗*^03:19	1 (0.001)	0 (0)
DRB1^*∗*^04:05	15 (0.012)	7 (0.015)	DQB1^*∗*^04:02	37 (0.029)	10 (0.021)
DRB1^*∗*^04:06	2 (0.002)	2 (0.004)	DQB1^*∗*^05:01	183 (0.144)	62 (0.131)
DRB1^*∗*^04:07	20 (0.016)	1 (0.002)	DQB1^*∗*^05:02	25 (0.02)	23 (0.049)^b^
DRB1^*∗*^04:08	0 (0)	2 (0.004)	DQB1^*∗*^05:03	36 (0.028)	9 (0.019)
DRB1^*∗*^07:01	210 (0.165)	80 (0.169)	DQB1^*∗*^06:01	14 (0.011)	9 (0.019)
DRB1^*∗*^08:01	29 (0.023)	10 (0.021)	DQB1^*∗*^06:02	116 (0.091)	41 (0.086)
DRB1^*∗*^08:02	3 (0.002)	1 (0.002)	DQB1^*∗*^06:03	92 (0.072)	33 (0.07)
DRB1^*∗*^08:03	2 (0.002)	0 (0)	DQB1^*∗*^06:04	36 (0.028)	18 (0.038)
DRB1^*∗*^08:04	2 (0.002)	0 (0)	DQB1^*∗*^06:09	12 (0.009)	4 (0.008)
DRB1^*∗*^08:06	3 (0.002)	0 (0)			
DRB1^*∗*^09:01	8 (0.006)	5 (0.011)			
DRB1^*∗*^10:01	25 (0.02)	8 (0.017)			
DRB1^*∗*^11:01	84 (0.066)	28 (0.059)			
DRB1^*∗*^11:02	16 (0.013)	5 (0.011)			
DRB1^*∗*^11:03	10 (0.008)	1 (0.002)			
DRB1^*∗*^11:04	39 (0.031)	22 (0.046)			
DRB1^*∗*^12:01	13 (0.01)	7 (0.015)			
DRB1^*∗*^13:01	94 (0.074)	34 (0.072)			
DRB1^*∗*^13:02	49 (0.039)	22 (0.046)			
DRB1^*∗*^13:03	21 (0.017)	6 (0.013)			
DRB1^*∗*^13:05	3 (0.002)	2 (0.004)			
DRB1^*∗*^14:01	32 (0.025)	9 (0.019)			
DRB1^*∗*^14:04	5 (0.004)	1 (0.002)			
DRB1^*∗*^15:01	121 (0.095)	44 (0.093)			
DRB1**∗**15:02	16 (0.013)	8 (0.017)			
DRB1^*∗*^15:03	1 (0.001)	0 (0)			
DRB1^*∗*^16:01	16 (0.013)	19 (0.04)^a^			

AF: allele frequency; *n*: number of observed alleles. ^a^Control vs. melanoma patients: *p*=0.001, Pc = 0.035, and OR = 3.28. ^b^Control vs. melanoma patients: *p*=0.001, Pc = 0.017, and OR = 2.54.

**Table 4:** Frequency of HLA-associated alleles in melanoma patients grouped according to clinical features.

	DRB1*∗*16:01				DQB1*∗*05:02			
	%	*p*	Pc	OR	%	*p*	Pc	OR
Control	16 (0.013)				25 (0.02)			
Breslow thickness								
≤1.5 mm	9 (0.031)	0.023	Not significant		11 (0.038)	0.058	Not significant	
>1.5 mm	10 (0.054)	0.001	0.035	4.46	12 (0.065)	0.001	0.017	3.44
Age at diagnosis								
≤58 years	10 (0.042)	0.001	0.035	3.41	13 (0.054)	0.002	0.034	2.86
>58 years	9 (0.038)	0.004	Not significant		10 (0.043)	0.031	Not significant	

## Data Availability

The data used to support the findings of this study are available from the authors upon request.
